# Genetic evaluation of the variants using MassARRAY in non-small cell lung cancer among North Indians

**DOI:** 10.1038/s41598-021-90742-1

**Published:** 2021-05-28

**Authors:** Gh. Rasool Bhat, Itty Sethi, Amrita Bhat, Sonali Verma, Divya Bakshi, Bhanu Sharma, Muddasser Nazir, Khursheed A. Dar, Deepak Abrol, Ruchi Shah, Rakesh Kumar

**Affiliations:** 1 Cancer Genetics Research Group, ICMR, Centre for Advanced Research, School of Biotechnology, Shri Mata Vaishno Devi University, Katra, J&K UT India; 2 Department of Obstetrics and Gynecology, Government Medical College Srinagar, Srinagar, India; 3 Chest Disease Hospital, Government Medical College, Srinagar, Srinagar, India; 4 Department of Radiotherapy, Government Medical College, Kathua, Jammu, India; 5 Department of Biotechnology, University of Kashmir, Srinagar, Jammu and Kashmir India

**Keywords:** Cancer, Genetics, Diseases, Medical research, Oncology, Risk factors

## Abstract

Lung cancer is genetically diverse and a major health burden. Non-small cell lung cancer (NSCLC) accounts for 80% of total lung cancer cases and 20% cases are Small cell lung cancer (SCLC). The present case–control association study focused on the cost effective high throughput genotyping using Agena MassARRAY matrix-assisted laser desorption/ionization-time of flight, mass spectrometry (MALDI-TOF) platform to analyze the genetic association of candidate genetic variants. We performed multiplex PCR and genotyped twelve single nucleotide polymorphisms (SNPs) in 723 samples (162 NSCLC cases and 592 healthy controls). These genetic variants were selected from literature for their association with various cancers worldwide and this is the first study from the region to examine these critically important genetic variants. With prospective case–control association study design, twelve variants from ten genes were evaluated. Amongst these six variants, *TCF21* (rs12190287), *ERCC1* (rs2298881, 11615), *ERCC5* (rs751402), *ARNTL* (rs4757151), *BRIP1* (rs4986764) showed significant association with NSCLC risk (*p* ≤ 0.003) in Jammu and Kashmir population. In-silico findings of these genetic variants showed remarkable functional roles that needs in-vitro validations. It is further anticipated that such case control studies will help us in understanding the missing heritability of non-small cell lung cancer.

## Introduction

Lung cancer, a genetically heterogeneous disease is one of the leading causes of cancer incidence and mortality. It accounts for ~ 2.1 million new lung cancer cases and 1.8 million deaths worldwide^1^. In India, lung cancer is the chief cause of cancer-related mortality in both men and women^2^ and its incidence is rising at an alarming rate accounting for 11.3% of all new cancers and 13.7% cancer associated death^3–5^. Among North Indian region, Union territory of Jammu and Kashmir (J&K) is at greater risk of death rate related to various cancers. The incidence of lung cancer and breast cancer is higher followed by esophageal cancer in Jammu region of J&K as reported by a recent study. The study on the Kashmir region revealed that gastric carcinoma was commonly occurring cancer followed by lung carcinoma (9%) in general^6^. Despite making several efforts to enhance the 5 year survival rate of lung cancer patients, it remains 15–20%, the lowest of all cancers^7^. Currently, candidate gene approach (CGA) and Genome wide association studies (GWAS) has confirmed to be significant tools in interpretation of genetic complexity and heterogeneity of these disorders through association studies. With the successive GWAS, over the recent past more than 60 genetic loci have been found to be linked with NSCLC risk. Genetic characterization of variants have attracted significant attention in current medical era as potential biomarkers for predicting disease susceptibility and therapeutic targets^8^.

With this background, the variants in genes, which are critically important in various biological pathways like DNA damage and repair, invasion, metastasis, autophagy, circadian rhythm, apoptosis and signaling processes like *TCF21, ERCC1, BRIP1, ARNTL, ERCC5, REV1, PIK3CA, CASC16, DDC, BCL2* were targeted*.* This is the first ever genomic study from the region targeting the critical genes involved in the pathophysiology of non-small cell lung cancer. It is noteworthy that such studies will provide the holistic view of genetic landscape of non-small cell lung cancer in population of Jammu and Kashmir, North India. With this perspective, we evaluated twelve genetic variants of ten genes that are critically important and were previously associated with various cancers including lung cancer.

## Results and discussion

Lung cancer is the major global health burden contributing for more than million death worldwide. Before the GWAS era, the identification and characterization of lung cancer loci has been quite limited. GWAS, transcriptome wide association study (TWAS) and CGA has proved to be significant approach in understanding the genetic complexity and heterogeneity of multifactorial disorders through association studies. Worldwide so far, more than 60 loci have been linked with lung cancer by GWAS and candidate gene approach. Nevertheless, these genes are linked with multiple lung cancer pathways^9^. Currently, various susceptibility genes encoding various enzymes involved in the activation, cell-cycle pathways, circadian rhythm pathways and DNA damage and repair caused by smoke as well as genes involved in inflammatory and apoptosis processes have been studied extensively. Insights about the genetic and molecular mechanism is precondition to improve the clinical management and progress into novel therapeutic interventions. In present study, we evaluated twelve genetic variants of ten genes that are critically important and were previously associated with various cancers including non-small cell lung cancer. These genetic variants were associated with many biological pathways like DNA damage and repair, signaling processes, cell cycle, autophagy, circadian rhythm, apoptosis etc. Clinical and various epidemiological parameters has been enlisted in Table 1. The population enrolled in this study was genotyped for twelve genetic variants of ten genes including *TCF21* (rs12190287), *ERCC1* (rs2298881, 11615), *ERCC5* (rs751402), *ARNTL* (rs4757151, rs1026071), *BRIP1* (rs4986764), *REV1* (rs3792152), *PIK3CA* (rs2699887), *CASC16* (rs3803662), *DDC* (rs2229080) and *BCL2* (rs1801018) as mentioned in Supplementary Table 1. Following quality control (QC) check, the finalized data set remained as twelve genetic variants that passes the quality control analyses and followed the HWE and further tested for their association with NSCLC. Among twelve genetic variants, six variants were found to be significantly associated with non-small cell lung cancer as shown in Table 2, however six variants didn’t show any association with lung cancer risk in the population of J&K North India as shown in Table 3. Moreover, these genetic variations may interfere with epigenomics, transcription factor binding sites^10–12^. The possible functional role of the variants using databases GTEx v.7, UCSC, HaploReg v4.1, HSF (v.3.1) and ESE v.3 was assessed^13,14^. The findings of each variant has been summarized below and described in Table 4 and Fig. 3.

**Table 1:** showing the clinical parameter distribution between non-small cell lung cancer patients and healthy controls from Jammu and Kashmir population.

S. no	Characteristic	Cases	Controls	*p* value
1	Age* (in years)	61.3 ± 9.5	52.6 ± 15.3	< 0.05
2	BMI **	22.3 ± 3.92	25.47 ± 5.16	< 0.05
3	Gender—Male	n = 133	n = 177	–
	Gender—Female	n = 29	n = 384	
4	**Smoking status**			
	Smokers	142	80	–
	Non smokers	20	481	
5	**Alcoholic status**			
	Alcoholic	72	55	–
	Non alcoholic	90	506	
6	**Guthka status**			
	Guthka	16	–	–
	No Guthka	146	–	
7	**Histological subtypes**			
	AC	100	–	–
	SCC	54	–	
	UDC	08	–	
8	**Metastasis**			
	Metastatic	35	–	–
	Non metastatic	127	–	
9	**Family history**			
	Yes	29	–	–
	No	112	–	
	Not available	21	–	

*AC* Adenocarcinoma, *SCC* Squamous cell carcinoma, *UDC* Large cell undifferentiated Carcinoma.

*Age in years and **BMI in kg/m^2^.

**Table 2:** Allelic, genotypic distribution and logistic regression analysis of significant variants of genes in our study.

Variant	rs12190287		rs751402		rs4986764		rs2298881		rs4757151		rs11615	
Gene W.R.T variant	*TCF21*		*ERCC5*		*BRIP1*		*ERCC1*		*ARNTL*		*ERCC1*	
Polymorphism	C/G		A/G		A/G		C/A		A/G		A/G	
Allele distribution	G	C	A	G	A	G	A	C	G	A	G	A
Cases	0.532	0.468	0.329	0.671	0.455	0.545	0.215	0.785	0.504	0.496	0.453	0.547
Controls	0.648	0.352	0.268	0.732	0.546	0.454	0.289	0.711	0.584	0.416	0.531	0.469
Odds ratio at 95% CI	1.62 (1.26–2.09)		1.34 (1.02–1.75)		1.44 (1.11–1.86)		0.67 (0.49–0.90)		1.38 (1.06–1.80)		1.36 (1.03–1.80)	
Total HWE	0.174		0.829		0.931		0.709		0.429		0.839	
Genotypic model	Dominant (CC + GC vs GG)		Dominant AA + AG vs GG)		Additive (GG vs AG vs AA)		Additive (AA vs AC vs CC)		Recessive (AA vs AG + GG)		Recessive (AA vs AG + GG)	
Odds ratio at 95% CI	1.85 (1.14–2.99)		1.46 (1.00–2.13)		1.47 (1.12–1.94)		0.66 (0.48–0.91)		2.12 (1.32–3.47)		1.96 (1.23–3.11)	
*p* value*	0.012		0.027		0.006		0.012		0.002		0.006	

*Adjusted with age, gender and BMI.

**Table 3:** Allelic and Genotypic distribution of the variants, which did not show significant association with NSCLC in population of J&K, North India.

Variant	rs3792152		rs2699887		rs3803662		rs2229080		rs1801018		rs1026071	
Gene W.R.T variant	*REV1*		*PIK3CA*		*CASC16*		*DDC*		*BCL2*		*ARNTL*	
Polymorphism	A/G		C/T		A/G		C/G		A/G		A/G	
Total HWE	0.65		1.00		0.068		0.076		0.48		0.26	
Allele distribution	G	A	C	T	A	G	C	G	A	G	A	G
Cases	0.51	0.49	0.86	0.11	0.28	0.72	0.39	0.61	0.56	0.44	0.69	0.31
Controls	0.46	0.54	0.82	0.12	0.26	0.74	0.40	0.60	0.57	0.43	0.68	0.32
Odds ratio at 95% CI	1.24 (0.96–1.59)		0.74 (0.52–1.05)		1.15 (0.85–1.54)		0.98 (0.75–1.28)		1.02 (0.79–1.31)		0.99 (0.75–1.31)	
*p* value	0.092		0.095		0.36		0.925		0.872		0.985

**Table 4:** Putative Role of the associated variants with NSCLC in JandK Population—North India using the information from the different online databases including GTEX, UCSC genome browser and HSF.

Variant	Allele Ref/Alt	eQTL gene	eQTL Tissue	eQTL sample size	eQTL NES	eQTL *p* value	eQTL m-value	Putative role (cis-eQTL) of variant	Regulatory role of variant (ENCODE and Haploreg data)	Splicing effect
rs12190287	C/G	*TCF21*	Lung	515	0.29	1.9E − 17	1	Significant and Up regulation	3_TxFlnk/3_PromD1/H3K4me1_Enh/H3K4me3_Pro/H3K27ac_Enh/H3K9ac_Pro/23_PromBiv	Creation of new site/ broken site for 9G8
rs751402	A/G	*ERCC5*	Lung	515	0.14	6.2E − 4	1	Significant and Up regulation	1_TssA/H3K4me3_Pro/H3K27ac_Enh/H3K9ac_Pro/H3K4me1_Enh/DNase	Creation of new site for Tra2-β/ broken site for SRp40
rs4986764	A/G	*BRIP1*	Lung	515	− 0.09	3.8E − 3	0.985	Significant and Down regulation	NA	Site broken for SRp40
rs2298881	A/C	*ERCC1*	Lung	515	− 0.36	2.4E − 15	1	Significant and Down regulation	1_TssA/3_PromD1/H3K4me1_Enh/H3K4me3_Pro/H3K27ac_Enh/H3K9ac_Pro DNase hypersensitive	Site broken for SRp40
rs4757151	A/G	*ARNTL/BTBD10*	Lung	515	0.002	0.9	0.04	Not Significant	H3K4me1_Enh	Creation of new site/broken site for SC35
rs11615	G/A	*ERCC1*	Lung	515	− 0.05	0.1	0.6	Not Significant	11_TxEnh3/H3K4me1_Enh/H3K27ac_Enh/6_EnhG	Creation of new site/ broken site for SF2/ASF (IgM/BRCA1), SF2/ASF and G98
		*CD3EAP*			− 0.14	4.8E − 7	1	Significant and down regulation		

*Represents risk allele in this study; NES—Normalized Effect Size in eQTL; m-value—posterior probability that effect exists in each tissue, ranges between 0 and 1; H3K27Ac_Enh—chemical modification (acetylation) of the histone proteins (H3) at lysine 27 and associated with transcriptional initiation and open chromatin structure (active enhancer); H3K4me3–chemical modification (methylation) of the histone proteins (H3) at lysine 4, marks promoters that are active or poised to be activated; H3K4me1—chemical modification (methylation) of the histone proteins (H3) at lysine 4 and is associated with enhancers, and downstream of transcription starts.; H3K9ac—chemical modification (acetylation) of the histone proteins (H3) at lysine 9 and is associated with transcriptional initiation and open chromatin structure; Enh—Enhancers; Pro—Promoters; TSSA—active transcription start site; TxReg—transcription regulatory; PromD1—promoter downstream TSS; TSSAFlk—Flanking TSS; 22PromP—poised promoter; EnhW1—weak enhancer; EnhA2—active enhancer 2; the H3K4me1/2/3 and H3K36me2/3 are linked with genomic region which actively transcribing and H3K9me3, H3K27me3 and H4K20me3 with non-transcribing region; ESE—Exonic Splicing Enhancers; SR—Serine-Arginine rich proteins; 9G8, SC35—SR splicing factor; SF2/ASF (IgM-BRCA1)—Serine-Arginine rich proteins.

### Genetic variants which showed significant association with non-small cell lung cancer in this study

**Figure 1:**
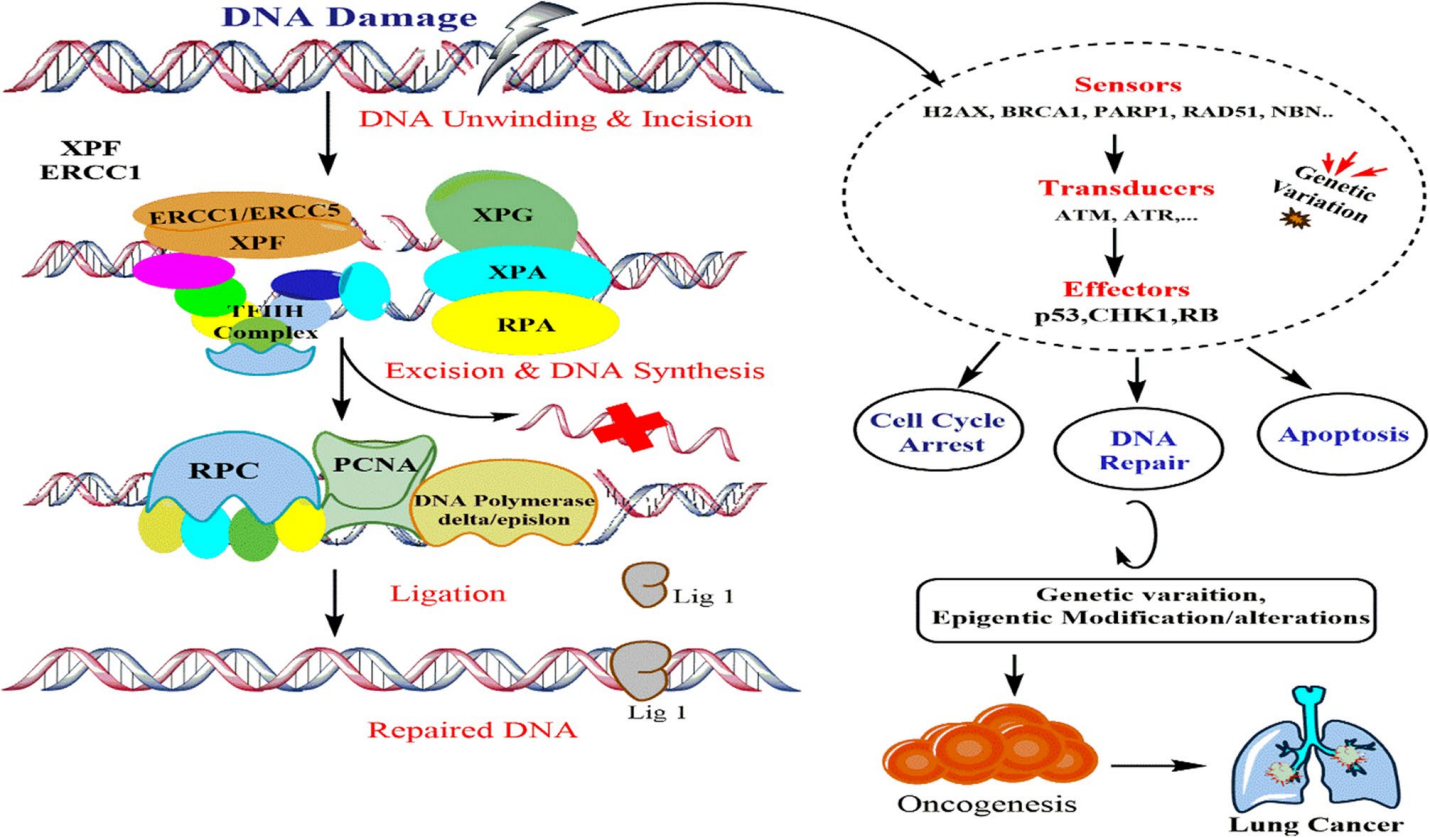
showing the DNA Repair process which include identifying DNA damage by DNA damage association proteins, then transducing damage signals to the cellular machinery, lastly, cell cycle arrest, however, the functional polymorphism in DNA repair genes can halt the repairing capability thus drives the cell towards oncogenesis and ultimately lung cancer (ChemBioDraw Ultra v.14.0.0.117).

Genetic variations in predominant genes, which maintain the genomic stability has been documented as a key factor for the individual risk to develop cancer. *ERCC1/ERCC5* genes are critically important factors in nucleotide excision repair pathway (NER). Excision repair cross complimentary group-1 (*ERCC1*) typically binds with XPF endonuclease (*ERCC4*) to form heterodimeric endonuclease (*XPF-ERCC1*) as shown in Fig. 1 during excision step at damaged site. This dimeric complex is also important interstrand crosslinks and homologous repair machinery, which activates the *RPC, PCNA*, DNA polymerase δ/ε followed by ligation step for repair process. Thus the functional variation/polymorphism in *ERCC1/ERCC5,* establishes the DNA repair capacity in the cell in order to maintain the genomic stability, may be a potential risk factor in the early process of oncogenesis as shown in Fig. 1. Various studies in such domains have been conducted in recent past to demonstrate the association of the genetic polymorphism and lung cancer risk^15–17^.

### rs11615

In this cases-control association study among various DNA repair genes and NSCLC risk in population of J&K, north India. The variant rs11615 is synonymous variant of the ERCC1 gene. In this study, the major allele (A) of variant rs11615 (A/G) demonstrated significantly increased risk for non-small lung cancer with an odds ratio (OR) of 1.96 (1.23–3.11 at 95% of CI) and *p* value of 0.006 (Table 2). The findings from the study are consistent with previous studies reported in meta-analysis of Asian/Caucasian pooled population^16^. Our study indicated that genetic variant rs11615 of *ERCC1* is a risk factor of NSCLC in Jammu & Kashmir population.

Furthermore, the findings of cis-eQTL analysis, the risk allele (A) is linked with down regulation of the expression of the gene in lungs (*p* value = 0.1 and normalized effect size (NES) = − 0.05). Since the gene is very critical in DNA repair process^17^, so the downregulation of gene might affect the repair efficiency. Moreover, the locus exhibited the existence for histone marks as (H3K4me1_Enh/H3K4me3_Pro/H3K27ac_Enh/H3K9ac_Pro) indicating promoter and transcription regulation, active transcription start site (TSS) promotor activity. Besides that in order to examine the consequence of this genetic variant on *ERCC1* gene using *insilco* approach. The widely used algorithms for the prediction of enhancer/silencer motifs by HSF demonstrated that rs11615 results in the broken site for SF2/ASF (IgM/BRCA1), SF2/ASF and creation of new sites. It was observed that variation in splicing factor binding of exonic splicing enhancer (ESE) intronic site signifies its vital role in epigenomics (Table 4 and Fig. 3).

### rs2298881

Another variant rs2298881 is an intronic variant of the *ERCC1* and was significantly associated with non-small cell lung cancer, but major allele (A) of variant rs2298881 (C/A) showed the protection against the NSCLC with an odds ratio (OR) of 0.66 (0.48–0.91 at 95% of CI) and *p* value of 0.012 (Table 2). The results are consistent with previous studies on meta-analysis suggesting rs2298881 is not a risk-associated polymorphism in lung cancer^16^.

Moreover, during the cis-eQTL analysis, it was observed that the risk allele (A) is related with down regulation of the gene in lungs (*p* value = 2.4E−15 and normalized effect size (NES) =  − 0.36). Since the gene is vital DNA repair process, so the downregulation of gene might affect the repair capacity. Furthermore, the locus exhibited the existence for histone marks as (H3K4me1_Enh/H3K4me3_Pro/H3K27ac_Enh/H3K9ac_Pro/DNase hypersensitive) suggesting promoter and transcription regulatory activity, active transcription start site (TSS) promotor activity. In order to examine the influence of this variant on *ERCC1* using insilco analysis. The prediction tools suggested that rs2298881 develop Site broken for SRp40. It was observed that alteration in splicing factor binding of exonic splicing enhancer (ESE) intronic site indicating its effect on epigenetic process (Table 4 and Fig. 3).

### rs751402

Variant rs751402 is 5´UTR variant of the *ERCC5*. In present study, the major allele (A) of rs751402 (A/G) exhibited significant association with non-small lung cancer risk with an odds ratio (OR) of 1.46 (1.00–2.13 at 95% of CI) and *p* value of 0.02 (Table 2). This variant has been extensively studied in different cancers (gastric, breast, salivary gland tumour) in different population groups^18–21^ including lung cancer^22^. The present study also indicated that genetic variant rs751402 is a risk factor of NSCLC in J&K population.

Cis-eQTL analysis demonstrated that the risk allele (A) is significantly related with up regulation of the gene in lungs (*p* value = 6.2E−4 and normalized effect size (NES) = 0.14). Since the gene is essential for DNA repair process, so the upregulation of gene might affect nucleotide excision repair pathway. Moreover, the region of interest exhibited the existence of histone marks as (H3K4me3_Pro/H3K27ac_Enh/H3K9ac_Pro/H3K4me1_Enh/DNase) signifying role in epigenetic regulation. *Insilco* approach also indicated that rs751402 results in creation of new site for Tra2-β/ broken site for SRp40. It was perceived that change in splicing factor binding of exonic splicing enhancer (ESE) intronic site may influence the physiology of the gene (Table 4 and Fig. 3).

**Figure 2:**
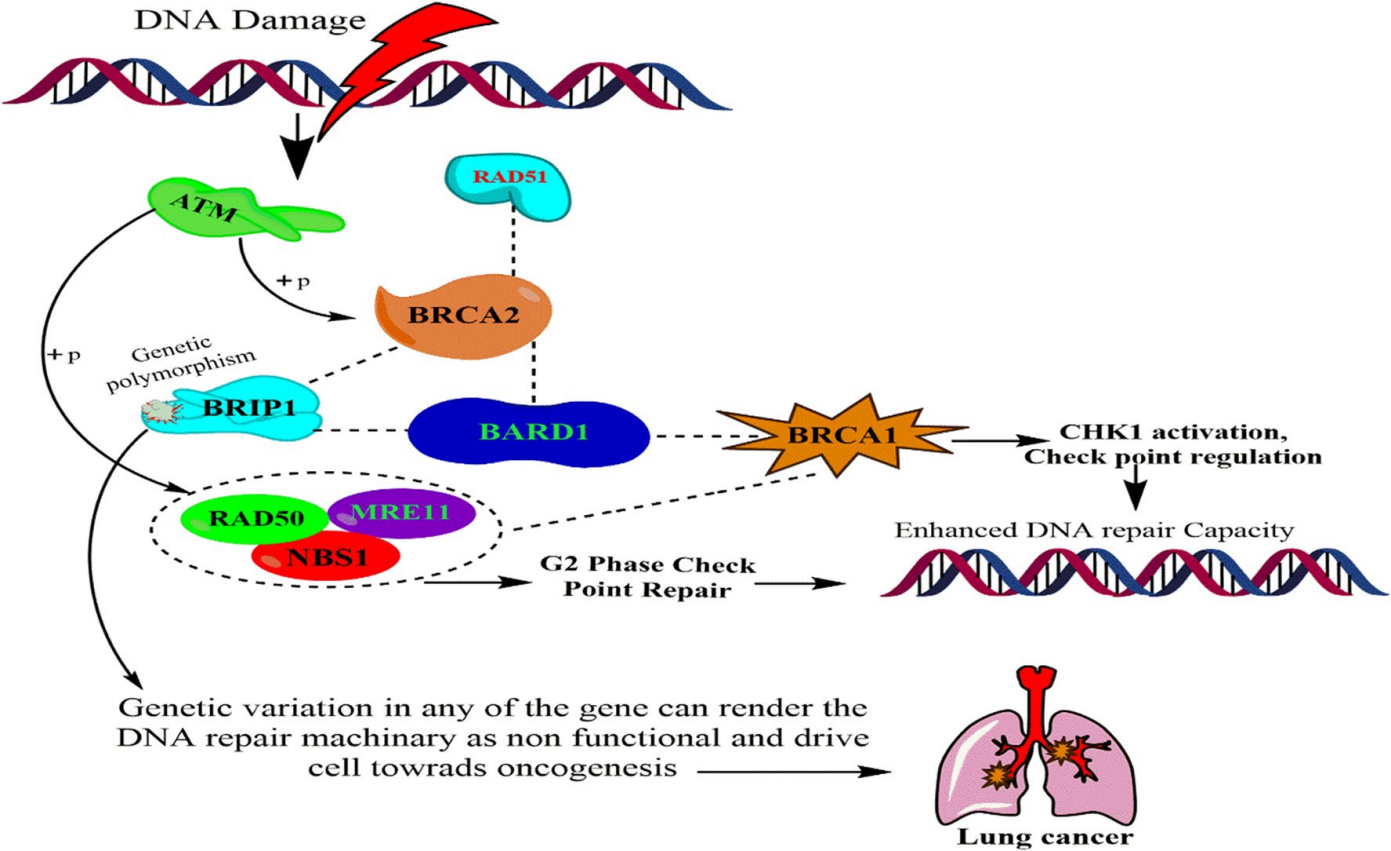
Showing the biological interaction and role of *BRIP1*. *BRIP1* (BRCA1 Interacting Protein C-Terminal Helicase 1) encodes a factor, which is an integral member of RecQ DEAH helicase family, which intercommunicate with repeats of BRCA type 1 (BRCA1). The composite complex is critical in normal double strand break repair processes (ChemBioDraw Ultra v.14.0.0.117).

The process of genomic instability is associated with earlier process of oncogenesis. Many essential genes maintain the genome stability and complexity by responding the DNA damage and repair machinery^23^. Among one such important gene is *BRIP1* (BRCA1 Interacting Protein C-Terminal Helicase 1) encodes a factor, which is an integral member of RecQ DEAH helicase family, which intercommunicate with repeats of BRCA type 1 (*BRCA1*). The composite complex is critical in normal double strand break repair processes. *BRIP1* encodes 1249 amino acid long protein that colocalizes with BRCA1 DNA damage site, and enhances to its DNA repair function^24^. During the DNA double strand break repair *BRCA2* interacts with *RAD51* resulting in *BRCA2/RAD51* complex. The complex colocalizes to damage induced foci where actual DNA repair process has to take place^25^. *BRIPI* is critically important in maintaining the genomic stability by regulating the GM1/2 checkpoints and *CHK1* activation as shown in Fig. 2.

### rs4986764

Variant rs4986764 is the missense variant of the BRIP1 gene. The study evaluated the genetic association of rs751402 with NSCLC risk in population of Jammu and Kashmir, North India. The major allele (A) of variant rs751402 (A/G) displayed significant association with non-small lung cancer risk with an odds ratio (OR) of 1.47 (1.12–1.94 at 95% of CI) and *p* value of 0.006 (Table 2). Various studies have demonstrated the effect of the genetic variation rs4986764 in *BRIP1* with multiple cancers including the non-small lung cancer^26–28^. Some studies demonstrated that genetic variation in any of associated genes result in reduced repair efficiency, which drives cell towards oncogenesis^26^. Thus, present study indicated that genetic variant rs4986764 (*BRIP1*) is a risk factor of non-small cell lung cancer in Jammu and Kashmir population, North India.

**Figure 3:**
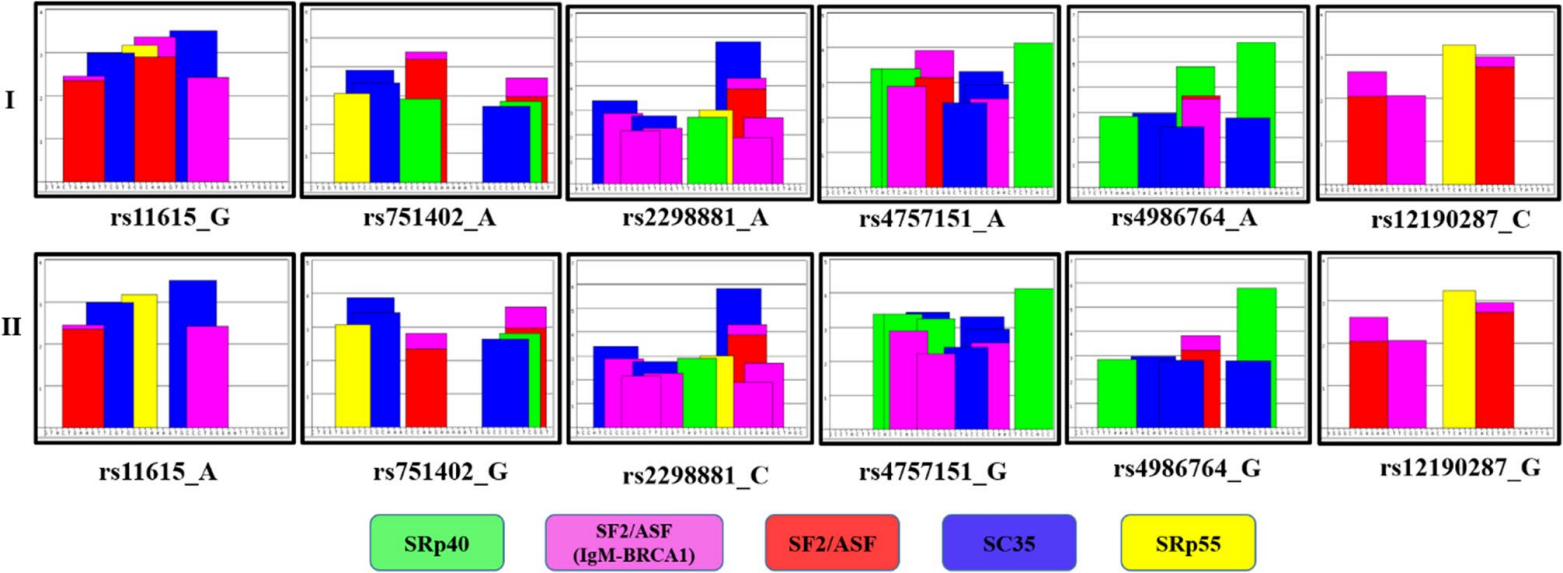
Effect of genetic variation on the Exonic Splicing Enhancers (ESEs) according to ESE prediction tool. ESE finder enables to recognize the potential ESE sites. The elevation of the colored bars represents the motif scores and the girth of the bars indicates the length of the motif. Bars in red, yellow, blue, purple and green indicate potential binding sites for Serine-Arginine (SR) proteins SF2/ASF, SRp55, SC35, SF2/ASF (IgM-BRCA1) and SRp40, respectively. Panel-I signifies the ESE sequence with the allele not posing risk in the population under study and panel-II denotes the ESE sequence with the risk allele in the studied population. From the figure, we can predict that there is a change in the potential ESE sites as can be seen from change in the bars (change in the potential splicing sites) that might increase the disease susceptibility (Human Splicing Finder (HSF) 3.1and ESE finder 3.0).

Cis-eQTL analysis advocated that risk allele (G) is significantly related with downregulation of the gene in lungs (*p* value = 3.8E−3 and normalized effect size (NES) = − 0.09). The said gene is the key component for DNA repair process^24^, so the downregulation of gene might critically effect the DNA repair pathway. Moreover, in order to examine the influence of this variant on *BRIP1* using insilco analysis. It was observed that rs4986764 results in site broken for SRp40. It was demonstrated that the alteration in splicing factor binding of exonic splicing enhancer (ESE) intronic site might disturb the regulation of gene (Table 4 and Fig. 3).

Transcription factor 21 (*TCF21*) belongs to helix loop helix (HLH) family of transcription factors, which have critical role in development of tissues of lung, heart and kidney. It harbor 3 exons associated with CpG islands (CpG1, CpG2 & CpG3). Higher rates of *TCF21* promoter hypermethylation processes have been observed in cancers of different origins, including lung cancer. The activation of *TCF21* by long ncRNA *TCF21* antisense RNA-inducing demethylation (TARID) by induction of promoter demethylation. Promoter of *TCF21* in third CpG guides the TARID transcription, thereby inducing the DNA demethylation (TET protein-dependent) resulting *TCF21* transcriptional activation and interaction of *TARID* to promoter of *TCF21*, which inducts GADD45A/TDG to base excision repair (BER) for demethylation processes^29^. A recent study on *TCF21* revealed the expression of *TCF21* in normal lung airways with the observation of aberrantly methylated and silenced in majority of non-small lung carcinomas^30^. Genetic variation rs12190287 can control *TCF21* expression and may function as a potent biomarker for genetic susceptibility to lung cancer.

### rs12190287

Genetic variant rs12190287 is 3´UTR variant of the *TCF21.* The allele (C), which is the major allele of variant rs12190287 (C/G) indicated significant association with non-small lung cancer risk with an odds ratio (OR) of 1.85 (1.14–2.99 at 95% of CI) and *p* value of 0.012 (Table 2). The same genetic variant was examined in Chinese GWAS for risk factor in many cancer including breast, osteosarcoma, renal cell carcinoma^31–33^. However, various studies have demonstrated the downregulation of *TCF21* in breast cancer, bladder cancer, and non-small cell lung cancer^30^. Although this genetic variant has not been evaluated for lung cancer risk in any of the population group in india. This study is the first study to evaluate rs12190287 with non small lung cancer risk. The findings from the study strongly advocated rs12190287 of *TCF21* is risk factor NSCLC in the J&K Population, North India with *p* = 0.012.

Analysis through cis-eQTL suggests that allele (C) (risk allele) is significantly linked with up regulation of the gene in lungs (*p* value = 1.9E−17 and normalized effect size (NES) = 0.29). Since the gene is essentially important in many biological processes, thus the upregulation of gene can affect these biological processes. Moreover, the locus exhibited the existence of histone marks as H3K4me1_Enh/ H3K4me3_Pro/H3K27ac_Enh/H3K9ac_Pro/23_PromBiv) suggesting important role in epigenetic regulation. In-silco approach also indicated that rs12190287 results in broken site for 9G8 and creation of new site. The change was also observed in splicing factor binding of exonic splicing enhancer (ESE) intronic site (Table 4 and Fig. 3).

### rs4757151 and rs1026071

Circadian rhythms pathways, which has been characterized in almost all living species and are controlled by circadian rhythm genes^34^. Disruption in either genes or pathways has been associated with many ailments like mood related disorders, depression, cardiovascular disease and cancer. The monitoring feedback loop of circadian rhythm consists of critical genes like *ARNTL, PER, CLOCK,* which function as an important regulators of transcription and translation process.

Genetic variant rs4757151 is an intronic variant of the *ARNTL.* The allele (C) (major allele) of variant rs4757151 (C/G) exhibited significant association with NSCLC risk with an odds ratio (OR) of 2.12 (1.32–3.47 at 95% of CI) and *p* value of 0.002 (Table 2). This variant has not been evaluated for the non-small cell lung cancer risk in any Indian population group and our results proved that rs4757151 of *ARNTL* is a risk factor for NSCLC in J&K population, North India. Furthermore, in order to examine the effect of this genetic variant on *ARNTL* using in-silco analysis by Human Splicing finder (HSF) and exonic splicing enhancers (ESE). The majority of the algorithms used for the prediction of enhancer/silencer motifs by HSF indicated that rs4757151 results in broken site for SC35 and creation of new site (Table 4 and Fig. 3). Moreover, other variant rs1026071 of same gene didn’t show any genetic association with NSCLC risk with an odds ratio (OR) of 0.99 (0.75–1.31 at 95% of CI) and *p* value of 0.985. (Table 3).

### Non-significant genetic variants with non-small cell lung cancer

Various studies have linked the *DDC* expression with multiple cancer^35^. The genetic variant rs2229080 of *DDC* revealed the null association with the gastric and esophageal cancer risk in J&K population^36^. We similarly evaluated the same variant in population of Jammu and Kashmir for lung cancer risk and couldn’t found the genetic association with an odds ratio (OR) of 0.98 (0.75–1.28 at 95% of CI) and *p* value of 0.925 (Table 3). Genetic polymorphism in *PIK3CA* has been observed in several types of cancer including non-small cell lung cancer. Moreover genetic variation rs2699887 in *PIK3CA* has been associated with the brain metastasis in non-small cell lung patients. The study also revealed that NSCLC patients with one variant in rs2699887 had double the risk of having the brain metastasis than those without the variant^37^. The same variant was targeted in population of Jammu and Kashmir for lung cancer risk but we failed to find genetic association of same variant with an odds ratio (OR) of 0.74 (0.52–1.05 at 95% of CI) and *p* value of 0.095 (Table 3). Genetic variant rs3803662 of Cancer Susceptibility Candidate 16 gene (*CASC16*) is located at 16q12.1 is an RNA gene. The variant rs3803662 did not show any genetic association with NSCLC risk in population of Jammu and Kashmir with an odds ratio (OR) of 1.15 (0.85–1.54 at 95% of CI) and *p* value of 0.36 (Table 3). This polymorphism has been extensively associated with breast cancer risk in Iranian, Caucasian, Asian population groups^38^. REV1 DNA Directed Polymerase (*REV1*) gene shares homology to Y-family DNA polymerases, and act as scaffold protein involved in translesion synthesis (TLS) of damaged DNA^39^. Genetic variant rs3792152 is an intronic variant of REVI gene. The variant did not show the genetic association with NSCLC risk in population of Jammu and Kashmir with an odds ratio (OR) of 1.24 (0.96–1.59 at 95% of CI) and *p* value of 0.092 (Table 3). Various studies have demonstrated the role of *BCL-2* in oncogenesis, neuro disorders, ischemia and autoimmune diseases etc. *BCL-2* overexpression is associated with various cancers like NSCLC, esophageal cancer, endometrial cancer, breast cancer, CLL, diffuse large B-cell lymphoma etc.^40,41^. Genetic variant rs3792152 is coding sequence variant of *BCL-2.* The variant did not show any significant association with NSCLC risk in population of Jammu and Kashmir with an odds ratio (OR) of 1.02 (0.79–1.31 at 95% of CI) and *p* value of 0.872 (Table 3), which is in consistent with male Chinese population^42^ and Asian^43^ population groups, wherein they fail to find association of variant rs1801018 with NSCLC risk. Furthermore, the interaction between the genetic variants were evaluated through the multifactor dimensionality reduction software (MDR) v3.0.2. The variants (attributes) connected with shortest lines show strongest synergetic effect. The results indicted the variant *BRIP1, ERCC5, ERCC1* are linked with red colored line thus suggesting the strong interaction and maximum synergetic effect among the genes as shown in supplementary Fig. 2a,b. Best fit model as shown in supplementary Fig. 3a,b suggests interaction effect for the associated variants with NSCLC in the studied population and revealed the strong interaction among the *BRIP1, ERCC5,* and *ERCC1* genes respectively.

## Conclusion

The recent advances in high throughput techniques and molecular characterization of cancer related single nucleotide variants for improving the therapeutic interventions has been challenging task for scientists and clinicians. The case control association studies identifying the role of these genetic variants proved to be fruitful in such arena.

The present study explored the association of twelve critical genetic variants involved in diverse biological processes and their plausible regulatory role. Out of twelve genetic variations, after applying the QC and HWE analysis, six variants *TCF21* (rs12190287), *ERCC1* (rs2298881, 11615), *ERCC5* (rs751402), *ARNTL* (rs4757151), *BRIP1* (rs4986764) showed strong significant association with non-small lung cancer in population of Jammu and Kashmir, North India with (OR = 1.46–2.12 and *p* value ≤ E10−3) while six variants *REV1* (rs3792152), *PIK3CA* (rs2699887), *CASC16* (rs3803662), *DDC* (rs2229080), *ARNTL* (rs1026071) and *BCL2* (rs1801018) variants did not showed any significant association with NSCLC risk. Our result revealed the complex genetic mechanism and highlighted the critical role of various genetic variants in the pathogenesis of non-small cell lung cancer. Moreover, all the statistically significant variants showed the role in epigenetic regulation and have potential effect in modulation of the gene expression of its own or neighboring gene that might be responsible for underlying etiology of non-small cell lung cancer. This is the first study from the northern region targeting the important cancer related genetic variants as the union territory of J&K is genetically less explored state. Such studies are lacking in the region.

This prelude study, which advocated the relationship of genetic variants with other cancers but not with non-small cell lung cancer and the variants which deviated from HWE warrants to be replicated on large sample cohorts. The finding from our study will enlighten our cognizance of inter-population variances in non-small cell lung cancer etiology and strengthens GWAS outcomes as well. Furthermore, these association studies if conducted on large sample size would help contributing towards fulfilling the gap of remaining unexplained heritability of non-small cell lung cancer to greater extent. Furthermore, the genetic variants targeted in the present study warrants the functional analysis in future studies.

## Materials and methods

### Ethical statement

The study design was following the Helsinki Declaration and was confirmed by the Institutional Ethics Review Board (IERB) of Shri Mata Vaishno Devi University (SMVDU) vide IERB Serial No: SMVDU/IERB/16/41. The participants were informed about the research objectives and a written informed consent in three local languages was acquired from all the subjects enrolled in the present study. It was confirmed that all the methods were performed following the relevant guidelines and regulations.

### Sampling

A total of 723 subjects, 162 NSCLC cases and 561 healthy controls were enrolled for the study after informed consent from the individuals. All cancer cases were histopathologically confirmed. Two milliliters of venous blood sample was collected from each participant in an EDTA vial. Epidemiological features were summed up in Table 1.

### DNA isolation

Genomic DNA was isolated from the blood samples using Qiagen DNA Isolation kit (Catalogue No. 51206). The quantity and quality control analysis of genomic DNA was performed by carrying out UV spectrophotometer (Eppendorf Biospectrometer®, Hamburg Germany) analysis and Gel electrophoresis respectively.

### Selection of variants and genotyping

In this study, we selected genetic variants which have been associated in non-small cell lung cancer through GWAS and replication studies using the CGA. Finally, a total of twelve genetic variants of ten genes were shortlisted. The details of genetic variants are discussed in supplementary Table 1. Genotyping was performed at Central MassARRAY facility at SMVDU on a high-throughput Agena MassARRAY platform (The MassARRAY® System by Agena Bioscience™, San Diego, CA)^44^. The list of primers provided in supplementary Table 2.

Sequenom Typer 4.0 software was used to analyse genotype calls as in supplementary Fig. 1. In order to exclude the call errors via spectrograms all genotype calls were cross checked. The subjects were left out from the study if the missing genotypes were higher than 10%. Those variants which deviated from the Hardy–Weinberg Equilibrium (HWE) (*p* value < 0.05) were also omitted from the study. The genotyping results were replicated in 10% of random samples and the concordance rate was 98.5%. In the reaction of 384 well plates, one positive and one negative control were added for quality check.

### Genotyping quality control and criteria

Following criteria was used for validation and acceptance of genotyping. Genetic variants (SNPs) having call rate > 90% were included for statistical analysis^45^. Hardy–Weinberg Equilibrium (HWE) among cases and controls were used for assessing the quality of genotypes after analysing data sets. Those variants which didn’t follow the HWE (*p* value < 0.05) were also omitted from the study.

### Statistical analysis

Statistical t-test was used to compare by comparing the clinical characteristics between cases and controls. Genotype data was analysed by using the PLINK v. 1.07^46^ and IBM SPSS statistics 20 software^47^. All the genetic variants were tested for Hardy–Weinberg equilibrium using chi-square test. The association of variants with non-small lung cancer risk was validated by binary logistic regression analysis adjusted for confounding factors like age, gender and Body Mass Index (BMI). The odds ratios (ORs) were calculated based on the risk allele observed in this study conducted. One way ANOVA was employed for comparison of clinical characteristics of different genotypes for each variant, adjusted for age and gender (Supplementary Table 3).

### Potential role of the variants

University of California Santa Cruz (UCSC) Genome Browser (https://genome.ucsc.edu) and GTEx portal (https://www.gtexportal.org) combined was used for expression Quantitative Trait Loci (eQTL) analysis of the variants. Furthermore, UCSC Genome Browser, Encyclopedia of DNA Elements (ENCODE) (V3) and HaploReg v4.1 database^13,48^ tools were employed for the analyzing the transcriptional regulatory role like histone modifications, DNase hypersentivity and binding sites for the transcription factor. Besides that the effect of variant on splicing was evaluated by using the web tool Human Spicing Finder (HSF) 3.1and ESE finder (3.0)^14,49^.

## Supplementary information

Supplementary Information.

## References

[1] Bray F (2018). Global cancer statistics 2018: GLOBOCAN estimates of incidence and mortality worldwide for 36 cancers in 185 countries. CA Cancer J. Clin..

[2] Hussain Aliya N (2010). The lung Robbin’s and Cotran’s pathologic basis of disease. Saunders Company.

[3] Malik PS, Raina V (2015). Lung cancer: prevalent trends & emerging concepts. Indian J. Med. Res..

[4] Ganesh B, Sushama S, Monika S, Suvarna P (2011). A case-control study of risk factors for lung cancer in Mumbai, India. Asian Pac. J. Cancer Prev..

[5] Behera D (2017). SC17.03 lung cancer in India: challenges and perspectives. J. Thorac. Oncol..

[6] Qurieshi, M. A. *et al.* Epidemiology of cancers in Kashmir, India: an analysis of hospital data. *Adv. Prev. Med. ***2016** (2016). 10.1155/2016/1896761 PMC4949346 27478644

[7] Shields PG (2002). Molecular epidemiology of smoking and lung cancer. Oncogene.

[8] Zhou CP (2014). Association analysis of colorectal cancer susceptibility variants with gastric cancer in a Chinese Han population. Genet. Mol. Res..

[9] Wang, L. *et al.* Cross-cancer pleiotropic analysis reveals novel susceptibility loci for lung cancer. *Front. Oncol. ***9**, 1942 (2020). 10.3389/fonc.2019.01492 PMC6974684 32010612

[10] Xue A (2018). Genome-wide association analyses identify 143 risk variants and putative regulatory mechanisms for type 2 diabetes. Nat. Commun..

[11] Seo S (2013). Functional analysis of deep intronic SNP rs13438494 in Intron 24 of PCLO gene. PLoS ONE.

[12] Esmaeili R (2018). Unique CD44 intronic SNP is associated with tumor grade in breast cancer: a case control study and in silico analysis. Cancer Cell Int..

[13] Ward LD, Kellis M (2016). HaploReg v4: systematic mining of putative causal variants, cell types, regulators and target genes for human complex traits and disease. Nucleic Acids Res..

[14] Cartegni L, Wang J, Zhu Z, Zhang MQ, Krainer AR (2003). ESEfinder: a web resource to identify exonic splicing enhancers. Nucleic Acids Res..

[15] Zhou W (2005). Gene-smoking interaction associations for the ERCC1 polymorphisms in the risk of lung cancer. Cancer Epidemiol. Prev. Biomark..

[16] Zhu, J. *et al.* Association studies of ERCC1 polymorphisms with lung cancer susceptibility: a systematic review and meta-analysis. *PloS One* **9**, e97616 (2014). 10.1371/journal.pone.0097616 PMC4026486 24841208

[17] Du L (2018). Association of DNA repair gene polymorphisms with the risk of radiation pneumonitis in lung cancer patients. Oncotarget.

[18] Duan Z (2012). Promoter polymorphisms in DNA repair gene ERCC5 and susceptibility to gastric cancer in Chinese. Gene.

[19] Zavras AI, Yoon AJ, Chen M-K, Lin C-W, Yang S-F (2012). Association between polymorphisms of DNA repair gene ERCC5 and oral squamous cell carcinoma. Oral. Surg. Oral. Med. Oral. Pathol. Oral. Radiol..

[20] Xue, M. *et al.* DNA repair gene polymorphisms in ERCC4 rs6498486 and ERCC5 rs751402 and risk of salivary gland tumors. *Shanghai J. Stomatol. ***22**, 438–442 (2013). 24100905

[21] Chen YZ (2016). Association between XPG polymorphisms and stomach cancer susceptibility in a Chinese population. J. Cell Mol. Med..

[22] Bai Y (2007). Sequence variations in DNA repair gene XPC is associated with lung cancer risk in a Chinese population: a case-control study. BMC Cancer.

[23] Risinger MA, Groden J (2004). Crosslinks and crosstalk: human cancer syndromes and DNA repair defects. Cancer Cell.

[24] Cantor SB (2001). BACH1, a novel helicase-like protein, interacts directly with BRCA1 and contributes to its DNA repair function. Cell.

[25] Liu Y, West SC (2001). Distinct functions of BRCA1 and BRCA2 in double-strand break repair. Breast Cancer Res..

[26] Waqar SN (2014). BRCAness in non-small cell lung cancer (NSCLC). J. Clin. Oncol..

[27] Liu D (2018). Four common polymorphisms of BRIP1 (rs2048718, rs4988344, rs4986764, and rs6504074) and cancer risk: evidence from 13,716 cancer patients and 15,590 cancer-free controls. Aging (Albany NY).

[28] Ma X (2013). BRIP1 variations analysis reveals their relative importance as genetic susceptibility factor for cervical cancer. Biochem. Biophys. Res. Commun..

[29] Jiang X, Yang Z (2018). Multiple biological functions of transcription factor 21 in the development of various cancers. OncoTargets Ther..

[30] Smith LT (2006). Epigenetic regulation of the tumor suppressor gene TCF21 on 6q23-q24 in lung and head and neck cancer. Proc. Natl. Acad. Sci..

[31] Gao X, Yang J, Wang M, Zhang J (2016). TCF21 genetic polymorphisms and breast cancer risk in Chinese women. Oncotarget.

[32] Jiang Z (2017). Transcription factor 21 (TCF21) rs12190287 Polymorphism is associated with osteosarcoma risk and outcomes in East Chinese population. Med. Sci. Monit..

[33] Ye Y (2012). Down-regulation of TCF21 is associated with poor survival in clear cell renal cell carcinoma. Neoplasma.

[34] Takahashi JS (2017). Transcriptional architecture of the mammalian circadian clock. Nat. Rev. Genet..

[35] O’Loughlin, J. *et al.* Genetic variants and early cigarette smoking and nicotine dependence phenotypes in adolescents. *PloS One* **9**, e115716 (2014). 10.1371/journal.pone.0115716 PMC4278712 25545355

[36] Malik MA, Gupta A, Zargar SA, Mittal B (2013). Role of genetic variants of deleted in colorectal carcinoma (DCC) polymorphisms and esophageal and gastric cancers risk in Kashmir Valley and meta-analysis. Tumor Biol..

[37] Li Q (2013). Associations between single-nucleotide polymorphisms in the PI3K–PTEN–AKT–mTOR pathway and increased risk of brain metastasis in patients with non–small cell lung cancer. Clin. Cancer Res..

[38] Tajbakhsh A (2019). Significant association of TOX3/LOC643714 locus-rs3803662 and breast cancer risk in a cohort of Iranian population. Mol. Biol. Rep..

[39] Dumstorf CA, Mukhopadhyay S, Krishnan E, Haribabu B, McGregor WG (2009). REV1 is implicated in the development of carcinogen-induced lung cancer. Mol. Cancer Res. MCR.

[40] Anagnostou VK (2010). High expression of BCL-2 predicts favorable outcome in non-small cell lung cancer patients with non squamous histology. BMC Cancer.

[41] Sánchez-Beato M, Sánchez-Aguilera A, Piris MA (2003). Cell cycle deregulation in B-cell lymphomas. Blood J. Am. Soc. Hematol..

[42] Xu P (2013). Genetic variation in BCL2 3'-UTR was associated with lung cancer risk and prognosis in male Chinese population. PLoS ONE.

[43] Yao Z (2017). Genetic polymorphisms of Bcl-2 promoter in cancer susceptibility and prognosis: a meta-analysis. Oncotarget.

[44] Gabriel S, Ziaugra L, Tabbaa D (2009). SNP genotyping using the Sequenom MassARRAY iPLEX platform. Curr. Protoc. Hum. Genet..

[45] Anderson CA (2010). Data quality control in genetic case-control association studies. Na.t Protoc..

[46] Purcell S (2007). PLINK: a tool set for whole-genome association and population-based linkage analyses. Am. J. Hum. Genet..

[47] Fortunato O (2014). Mir-660 is downregulated in lung cancer patients and its replacement inhibits lung tumorigenesis by targeting MDM2-p53 interaction. Cell Death Dis..

[48] Ward LD, Kellis M (2012). HaploReg: a resource for exploring chromatin states, conservation, and regulatory motif alterations within sets of genetically linked variants. Nucleic Acids Res..

[49] Desmet F-O (2009). Human Splicing Finder: an online bioinformatics tool to predict splicing signals. Nucleic Acids Res..

