# Supplementary material for: Genetic evaluation of the variants using MassARRAY in non-small cell lung cancer among North Indians
Source: Sci Rep. 2021 May 28;11:11291. doi: 10.1038/s41598-021-90742-1 (PMC8163781; doi:10.1038/s41598-021-90742-1)
Supplement: Supplementary file 1 — Supplementary Information. [file 41598_2021_90742_MOESM1_ESM.docx]

**Genetic evaluation of the variants using MassARRAY in non-small cell lung cancer among North Indians**

**Gh. Rasool Bhat^1^, Itty Sethi^1^, Amrita Bhat^1^, Sonali Verma^1^, Divya Bakshi^1^, Bhanu Sharma^1^ Muddasser Nazir^2^, Khursheed A Dar^3^, Deepak Abrol^4^**, **Ruchi Shah^1^, Rakesh Kumar^*1^**

^1^ Cancer Genetics Research Group, School of Biotechnology, Shri Mata Vaishno Devi University, Katra

^2^ Department of Obstetrics & Gynaecology, Govt. Medical College Srinagar

^3^ Chest Disease Hospital, Government Medical College, Srinagar

^4^ Department of Radiotherapy, Government Medical College, Kathua.

**^*1^Corresponding Author**

**Dr. Rakesh Kumar, Assistant Professor,**

**School of Biotechnology** and

**Coordinator:** Cancer Genetics Research Group &

**Chief Coordinator**: ICMR, Centre for Advanced Research

School of Biotechnology, SMVDU, Katra, J&K UT, India

**Mobile: +919419279629. Ext. 2288**

**Email:** [**drrakeshthusoo@gmail.com**](mailto:drrakeshthusoo@gmail.com)

**And Dr. Ruchi Shah**

Department of Biotechnology

University of Kashmir, Jammu and Kashmir, India

[**scientistdobt@gmail.com**](mailto:scientistdobt@gmail.com)

**Supplementary Tables.**

**Supplementary Table 1:** Details of the variants selected for the study of non-small cell lung cancer in Jammu and Kashmir population

| **Gene** | **SNP** | **Chromosome: position** | **Location of the Variant w.r.t Gene** | **Function of Gene** |
| --- | --- | --- | --- | --- |
|  |  |  |  |  |
| ***TCF21*** | rs12190287 | 6:133893387 | UTR variant 3 prime | TCF21 encodes a transcription factor of the basic helix-loop-helix family. This gene include protein dimerization activity and proximal promoter DNA-binding transcription repressor activity, RNA polymerase II-specific. |
| ***ERCC5*** | rs751402 | 13:102845848 | 5 Prime UTR Variant | ERCC5 gene encodes a single-strand specific DNA endonuclease that makes the 3' incision in DNA excision repair following UV-induced damage. The protein may also function in other cellular processes, including RNA polymerase II transcription, and transcription-coupled DNA repair. |
| ***BRIP1*** | rs4986764 | 17:61685986 | Missense Variant | BRIP1 protein encoded by this gene is a member of the RecQ DEAH helicase family and interacts with the BRCT repeats of breast cancer, type 1 (BRCA1). This gene may be a target of germline cancer-inducing mutations. |
| ***ERCC1*** | rs2298881 | 19:45423658 | Intron Variant | ERCC1 gene functions in the nucleotide excision repair pathway and is required for the repair of DNA lesions. Genetic polymorphisms that alter expression of this gene may play a role in carcinogenesis. |
|  | rs11615 | 19:45420395 | Synonymous Variant |  |
| ***ARNTL*** | rs4757151 | 11:13370666 | Intron Variant | The protein encoded by this gene is a basic helix-loop-helix protein that forms a heterodimer with CLOCK. PER and CRY proteins heterodimerize and repress their own transcription by interacting in a feedback loop with CLOCK/ARNTL complexes. |
|  | rs1026071 | 11:13343165 | Intron Variant |  |
| ***REV1*** | rs3792152 | 2:99415089 | Intron Variant | REV1 gene shares homology to Y-family DNA polymerases, and act as scaffold protein involved in translesion synthesis (TLS) of damaged DNA |
| ***PIK3CA*** | rs2699887 | 3:179148620 | Intron Variant | PIK3CA is an integral member of PI3K–PTEN–AKT–mTOR pathway. This pathway is critical in the regulation of multiple cellular processes like cell survival, proliferation, adhesion and invasion. This pathway is balancer between cell growth and death. |
| ***CASC16*** | rs3803662 | 16:52552429 | Non Coding Transcript Variant | Cancer Susceptibility 16 (CASC16) is an RNA Gene, and is commonly associated with the lncRNA class. |
| ***DDC*** | rs2229080 | 18:52906232 | Missense  Variant | Dopa decarboxylase (DDC) gene encodes a principal component responsible for synthesis of dopamine and serotonin. |
| ***BCL2*** | rs1801018 | 18:63318646 | Synonymous  Variant | B cell lymphoma-2 (*BCL-2*) is mainly located in outer membrane of mitochondria and is an important apoptotic inhibitor with well-known carcinogenic potential and is studied for chemoreistance by activating anti apoptotic mechanism |

**Supplementary Table 2:** List of variants and their primer and probe (UEP) sequence.

| **Variant** | **Gene** | **First Primer Sequence** | **Second Primer Sequence** | **UEP Sequence** |
| --- | --- | --- | --- | --- |
| rs12190287 | ***TCF21*** | ACGTTGGATGTGGAAGGGTATCCTGACATC | ACGTTGGATGATTCTCCAAGGGCTGAGAAC | GCAAATAGACAGGTGGATGAA |
| rs751402 | ***ERCC5*** | ACGTTGGATGGTATTAGACGGAAACCGAGC | ACGTTGGATGAAACAGCCAGAAGATGTCCC | cGCGGGCCCATTTTTC |
| rs4986764 | ***BRIP1*** | ACGTTGGATGATAGATGACTTGCTGCTTCC | ACGTTGGATGGGACAATGAGTCTACACTTG | ACCTCTTTAAAGTACAGTACC |
| rs2298881 | ***ERCC1*** | ACGTTGGATGATTCTATTGGCTCCGTCCCC | ACGTTGGATGAGAGATGGACAAGGCCAGG | gtcaCCACCATCCCCCGCCTTCCGTT |
| rs11615 | ***ERCC1*** | ACGTTGGATGATAGTCGGGAATTACGTCGC | ACGTTGGATGTTGATGGCTTCTGCCCTTCG | TGAAGTTCGTGCGCAA |
| rs4757151 | ***ARNTL*** | ACGTTGGATGGTTTGTCCCTTTCCATGCTG | ACGTTGGATGATGAGACCTGGTGAGAGTTG | ggcgACTTTCACTCAGCTCC |
| rs1026071 | ***ARNTL*** | ACGTTGGATGACAGGCTAAATGTTGTGCCC | ACGTTGGATGTGTATGAGTGCCTAGGGTTC | TGCCTAGGGTTCAAATCT |
| rs3792152 | ***REV1*** | ACGTTGGATGGGAATGAAATGGCCTGAACC | ACGTTGGATGCCACTCAATAGGAGTTGGAG | gaggTGGAGGTACACCACA |
| rs2699887 | ***PIK3CA*** | ACGTTGGATGTGGGACCCGATGCGGTTAGA | ACGTTGGATGATTCCCACCGCACCCGCTA | gGTGAGTAGAGCGCGGA |
| rs3803662 | ***CASC16*** | ACGTTGGATGTTTCTTCGCAAATGGGTGGG | ACGTTGGATGTTTTCTCTCCTTAATGCCTC | ATGCCTCTATAGCTGTC |
| rs2229080 | ***DDC*** | ACGTTGGATGTCTTGCCCTCTGGAGCATTG | ACGTTGGATGGCTGAGCATCGGTAAATTCC | aaataTGGAGCATTGCAGATCAGC |
| rs1801018 | ***BCL2*** | ACGTTGGATGGTACTTCATCACTATCTCCC | ACGTTGGATGGTTGCTTTTCCTCTGGGAAG | cctaCCGGTTATCGTACCC |

**Supplementary Table 3:** One way ANOVA of significant Variants with quantitative traits.

| **Variant** | **rs12190287** | | | **rs751402** | | | **rs4986764** | | | **rs2298881** | | | **rs11615** | | | **rs4757151** | | |
| --- | --- | --- | --- | --- | --- | --- | --- | --- | --- | --- | --- | --- | --- | --- | --- | --- | --- | --- |
| **Gene** | ***TCF21*** | | | ***ERCC5*** | | | ***BRIP1*** | | | ***ERCC1*** | | | ***ERCC1*** | | | ***ARNTL*** | | |
|  | **MEAN±SE** | | | **MEAN±SE** | | | **MEAN±SE** | | | **MEAN±SE** | | | **MEAN±SE** | | | **MEAN±SE** | | |
| **Genotype** | **GG** | **GC** | **CC** | **GG** | **AG** | **AA** | **GG** | **AG** | **AA** | **CC** | **AC** | **AA** | **GG** | **AG** | **AA** | **GG** | **AG** | **AA** |
| **BMI** | 22.3  ±3.7 | 22.6  ±4.3 | 21.4  ±2.8 | 21.9  ±4.1 | 22.9  ±3.8 | 21.6  ±3.7 | 21.6 ±2.8 | 23.2  ±4.3 | 21.6  ±3.9 | 22.2  ±3.8 | 22.2  ±3.9 | 25.5  ±4.5 | 22.8  ±3.9 | 21.9  ±4.1 | 22.5  ±4.0 | 21.7  ±3.9 | 22.7  ±3.7 | 21.5  ±3.7 |
| **P-value** | **0.32** | | | **0.21** | | | **0.04** | | | **0.14** | | | **0.57** | | | **0.20** | | |
| **Age of onset** | 57.9±9.6 | 62.4 ±9.6 | 59.4  ±9.5 | 61.5  ±8.6 | 59.9  ±11.0 | 62.2  ±9.4 | 61.9  ±9.62 | 60.5  ±10.4 | 60.4  ±9.4 | 60.5  ±9.6 | 60.9  ±10.3 | 67.1  ±8.2 | 60.4  ±9.6 | 61.5  ±9.7 | 58.7  ±9.7 | 58.9  ±9.7 | 61.6  ±9.6 | 59.3  ±9.7 |
| **P-value** | **0.037** | | | **0.53** | | | **0.74** | | | **0.28** | | | **0.35** | | | **0.29** | | |
| **Smoking Status** | 12.3±7.3 | 13.4  ±7.6 | 12.7  ±7.3 | 23.1  ±9.5 | 21.4  ±9.5 | 21.4  ±10.4 | 24.7  ±11.9 | 21.7  ±8.9 | 20.7  ±8.5 | 22.7  ±9.7 | 21.2  ±8.8 | 25.0  ±16.2 | 20.38 ±9.3 | 23.14  ±9.5 | 21.50  ±9.5 | 20.8  ±9.5 | 23.5  ±9.5 | 22.4  ±9.4 |
| **P-value** | **0.69** | | | **0.55** | | | **0.16** | | | **0.55** | | | **0.43** | | | **0.40** | | |
| **Alcoholic Status** | 20.8±8.5 | 22.2  ±9.9 | 22.5  ±8.5 | 13.2  ±7.2 | 12.3  ±7.9 | 18.3  ±2.5 | 12.1  ±8.6 | 14.4  ±6.8 | 21.2 ±7.2 | 12.3  ±7.4 | 15.2  ±7.3 | 14.0  ±11.3 | 11.86  ±7.7 | 14.07  ±7.9 | 12.30  ±5.8 | 16.1  ±7.3 | 13.1  ±7.4 | 16.1  ±7.4 |
| **P-value** | **0.86** | | | **0.21** | | | **0.47** | | | **0.32** | | | **0.62** | | | **0.19** | | |


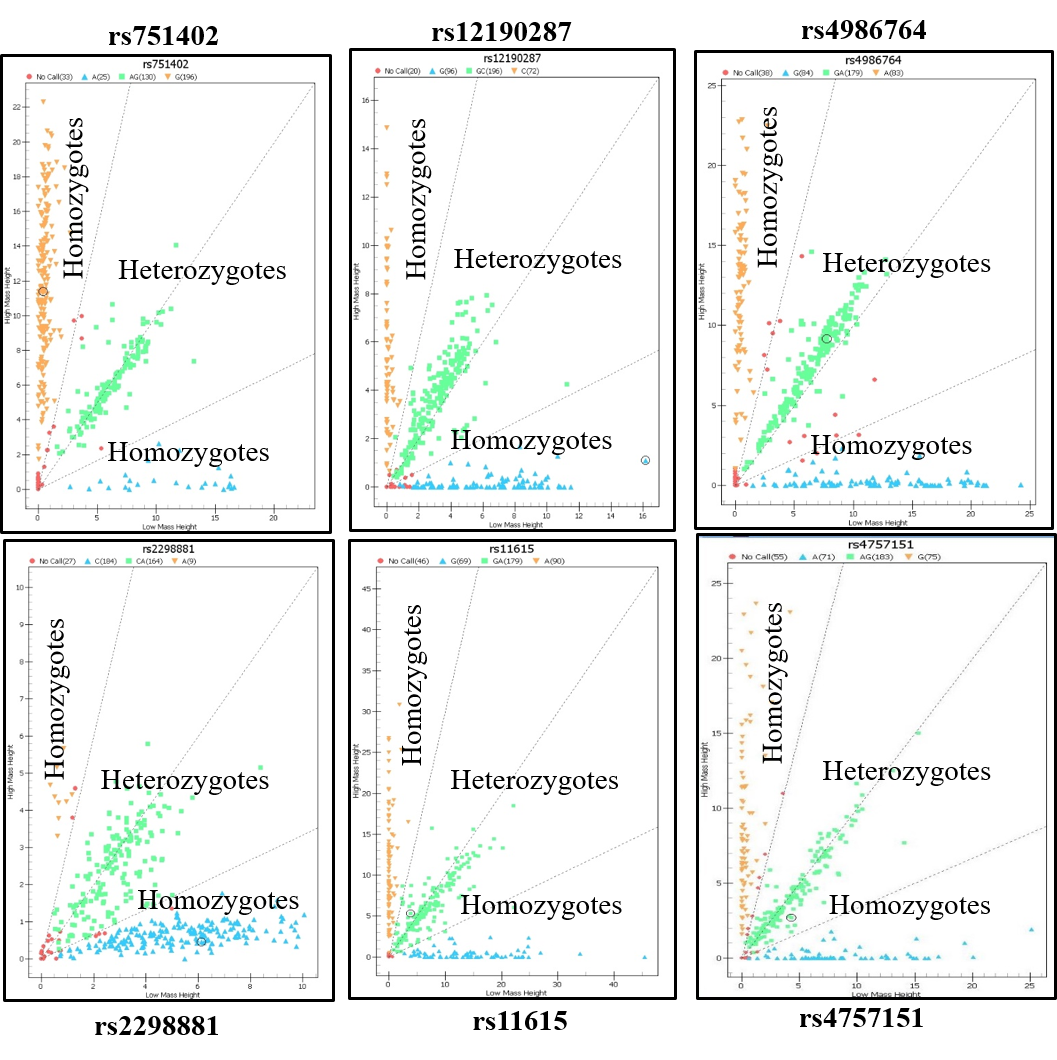
**Supplementary Figures**

**Supplementary figure 1:** Representative image of the MassARRAY Cluster Plot obtained after genotyping. Cluster plot of associated variants *ERCC5* (rs751402), *TCF21* (rs12190287), *BRIP1* (rs4986764), *ERCC1* (rs2298881, 11615), *ARNTL* (rs4757151) demonstrating the presence of homozygotes and heterozygotes in each variant (Typer 4.0).


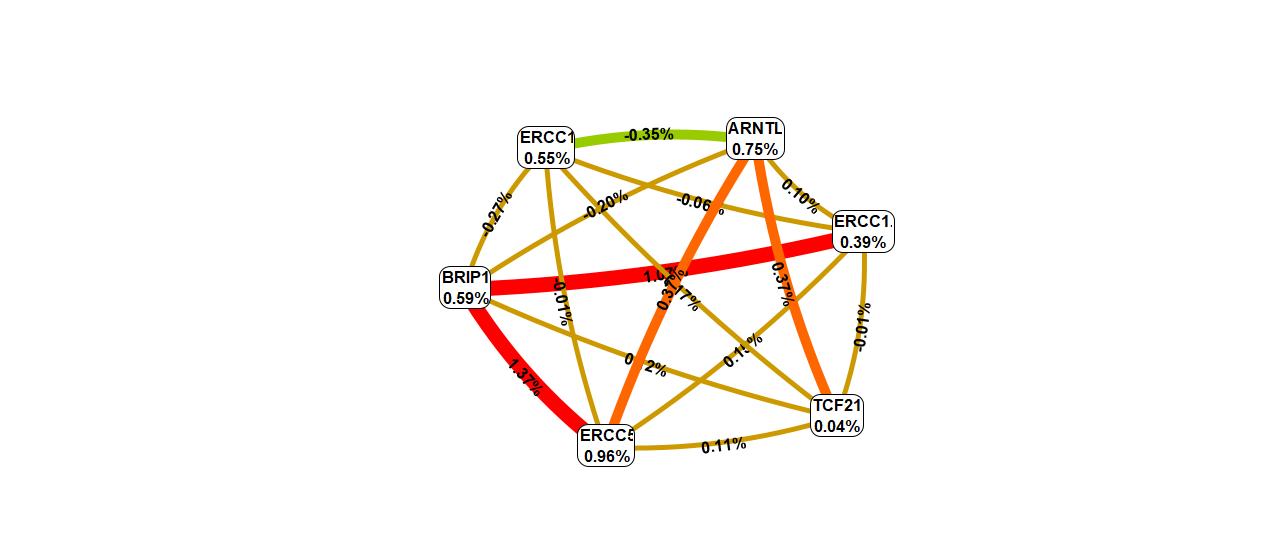


**Supplementary figure 2a: Illustration of Interaction Circle Graph for the Associated Variants with NSCLC in the Studied Population.** The interaction effect interpreted through the line colors. Red colour indicates highly synergetic effect, Orange colour indicates moderate synergetic effect, Light brown colour indicates additive effect and mainly independent effect and Blue and Green colour indicate no interaction or redundancy (Multifactor Dimensionality Reduction (MDR) v3.0.2).


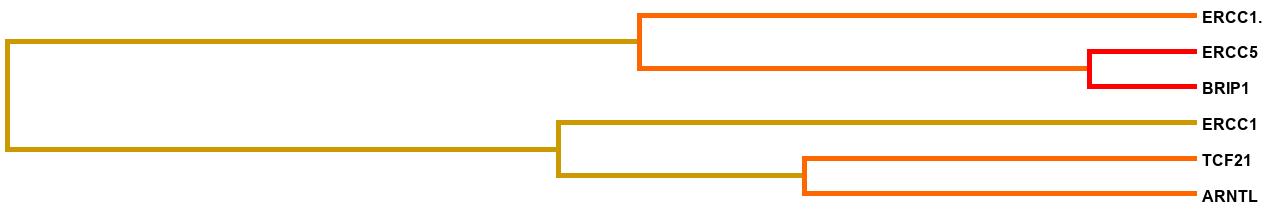


**Supplementary figure 2b: Illustration of dendogram for the associated variants with NSCLC in the studied population.** The interaction effect interpreted through the line colors and distance of lines. Red colour indicates highly synergetic effect, Orange colour indicates moderate synergetic effect, and Light brown colour indicates additive effect and mainly independent effect. The variants (attributes) connected with shortest lines show strongest synergetic effect (Multifactor Dimensionality Reduction (MDR) v3.0.2).


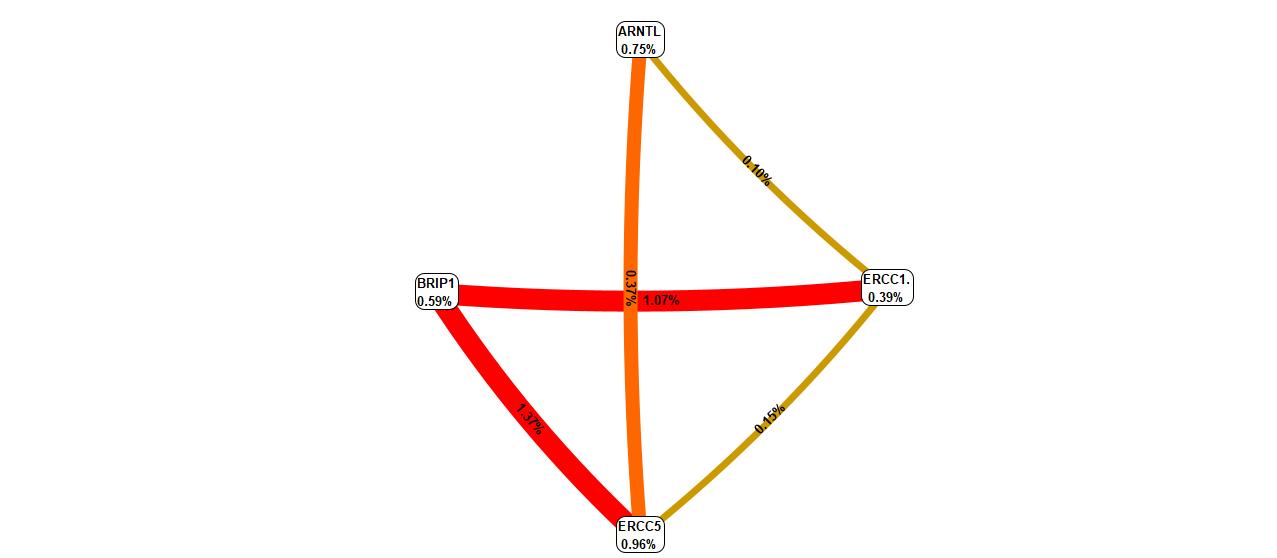


**Supplementary figure 3a: Representation of Best Fit Model of Interaction Effect for the Associated Variants with NSCLC in the Studied Population.** The interaction effect interpreted through the line colors. Red colour indicates highly synergetic effect between *ERCC5, BRIP1* and *ERCC1*and Orange colour indicates moderate synergetic effect between *ERCC1* and *ARNTL.* Light brown colour indicates additive effect between *ERCC5, ERCC1* and *ARNTL* (Multifactor Dimensionality Reduction (MDR) v3.0.2).


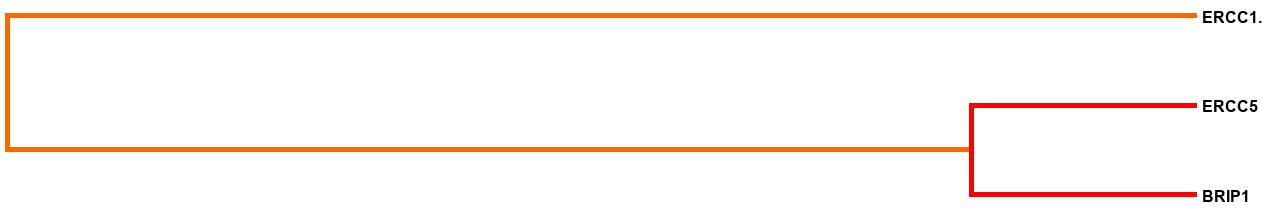


**Supplementary figure 3b: Representation of best fit model for dendogram of interaction effect for the associated variants with NSCLC in the studied population.** The interaction effect interpreted through the line colors. Red colour indicates highly synergetic effect i.e. between *BRIP1* and *ERCC5* and Orange colour indicates moderate synergetic effect of *ERCC1*. The variants (attributes) connected with shortest lines show strongest synergy effect (Multifactor Dimensionality Reduction (MDR) v3.0.2).
